# Are Some of the Cigar Warnings Mandated in the U.S. More Believable Than Others?

**DOI:** 10.3390/ijerph14111370

**Published:** 2017-11-10

**Authors:** Kristen L. Jarman, Sarah D. Kowitt, Jennifer Cornacchione Ross, Adam O. Goldstein

**Affiliations:** 1 Lineberger Comprehensive Cancer Center, University of North Carolina, Chapel Hill, NC 27599, USA; adam_goldstein@med.unc.edu; 2 Department of Health Behavior, Gillings School of Global Public Health, University of North Carolina, Chapel Hill, NC 27599, USA; kowitt@email.unc.edu; 3 Department of Social Sciences & Health Policy, Wake Forest School of Medicine, Winston Salem, NC 27157, USA; jcornacc@wakehealth.edu; 4 Department of Family Medicine, University of North Carolina, Chapel Hill, NC 27599, USA

**Keywords:** cigars, warnings, tobacco

## Abstract

*Background*: Text warnings are mandated on cigars sold in the United States (U.S.), however little published research has examined effectiveness of cigar warnings. This is the first study examining the believability of cigar warnings among adults in the U.S. *Methods*: Adults in the U.S. (*n* = 5014) were randomized in a phone survey to receive one of three cigar-specific mandated warning messages (“Cigar smoking can cause cancers of the mouth and throat, even if you do not inhale”, “Cigar smoking can cause lung cancer and heart disease”, and “Cigars are not a safe alternative to cigarettes”) with one of four warning sources (no source, Surgeon General, CDC (Centers for Disease Control and Prevention), FDA (Food and Drug Administration)). *Results*: Most adults found the cigar warnings very believable (66.9%). Weighted logistic regression results indicate that the message “Cigar smoking can cause lung cancer and heart disease” was associated with higher odds of being very believable (AOR: 2.05, 95% CI: 1.55, 2.70) and the message “Cigars are not a safe alternative to cigarettes” was associated with lower odds of being very believable (AOR: 0.71, 95% CI: 0.55, 0.92) compared to the message “Cigar smoking can cause cancers of the mouth and throat, even if you do not inhale”. Warning source had no impact on believability. *Conclusions*: We tested three of the currently mandated cigar warnings in the U.S. and found significant differences in believability between them. Further research on cigar warnings may improve communication to the public on cigar health risks, ultimately preventing uptake of cigars and promoting cessation among cigar users.

## 1. Introduction

More than one third of adults in the United States (U.S.) has used a cigar in their lifetime, and 5% have used a cigar in the past month [1]. Over the past 20 years, cigar use has increased [2] concurrent with a decrease in the use of cigarettes [3]. Two percent of adults use cigars on some days or every day, with higher rates among men, young adults (18–25), and those that identify as Black non-Hispanic or other non-Hispanic [4]. Most cigars sold in the U.S. fit into one of three categories, Large Cigars, Cigarillos, and Little Cigars [5], in this manuscript we use the term “cigar” to refer to all three of these product types. A recent analysis estimated that more than 140,000 years of potential life loss, and 9000 premature deaths were attributable to regular use of cigars in 2010, with years of life lost accounting for $23 billion in lost economic value [6].

In 2001 the Federal Trade Commission (FTC) issued rules for cigar packaging, including five rotating, mandatory warnings, three of which specifically mention cigars [7]. Regulation of some tobacco products was delegated to the Food and Drug Administration (FDA) in 2009, under the Family Smoking Prevention and Tobacco Control Act (FSPTCA) [8,9]. In May 2016, the FDA issued a final “deeming” rule, extending their authority to all tobacco products, including cigars [10,11]. The deeming regulation requires six rotating warnings, one of which includes an optional alternative warning (Table 1) [10,11,12]. Although four of the currently mandated warning statements are specific to cigars (1, 2, 3 and 5a), three are more broadly focused on tobacco products in general (4, 5b and 6), warning labels addressing addictiveness similar to number 6 are required on many other tobacco products [11].

To date, no experimental studies have addressed the impact of cigar warnings among adults; the extant literature focuses on cigarette warnings or involves qualitative designs [13,14]. A review of warning messages on tobacco products found that the impact of warnings depends on the design and size of the warning, and warnings that evoke a negative emotional reaction can increase intention to quit, among other outcomes [13]. This review found no relevant studies on cigar warnings, and called for more research on tobacco products other than cigarettes [13]. A more recent systematic review of health communication for non-cigarette tobacco products [15] revealed only two studies that assessed messaging for cigar products, with only one study, which was qualitative, that assessed cigar warnings [15]. Thus, we know little about effective messaging, including for warnings, for cigars. A recently published study examined believability of cigar warnings among adolescents (age 13–17) in the U.S., but this topic has not been addressed in adults, who are the main users of cigar products [16]. This lack of research coincides with data showing that from 2000 to 2015, while cigarette consumption decreased 39%, consumption of cigars among adults increased 85% [17]. Research specific to cigar warnings is also essential for other reasons, including: (1) cigar users have different demographic and consumption profiles when compared to cigarette users, and (2) evidence shows that cigar users misperceive that cigars are less harmful than cigarettes, despite having similar health effects [14]. 

Previous research in health communication and tobacco control has indicated that the source of a warning message may impact how the message is received [18], with a highly credible source being more effective at changing health behavior intentions [19]. Another study found that when a message was attributed to a highly credible source, when compared to a source with medium or low credibility, participants were more likely to trust and agree with the information [20]. However, no previous study has examined the impact of warning source on cigar warnings among adults. Of the required cigar warnings, only the optional alternative warning is attributed to a source, the Surgeon General [10,11].

Finally, believability has been demonstrated to be a metric of warning label impact, and may be a mediator in overall warning effectiveness [21,22,23]. A previous experimental study demonstrated that warning believability was associated with decreased desire to smoke, increased feelings toward quitting, and heightened risk perceptions of cigarettes [22]. Differences in believability between warnings may be an indication that more messaging, both in the form of communication campaigns and warnings, should be done to alleviate misconceptions about the health risks of cigar smoking. For instance, since many users perceive cigars to be less risky than cigarettes, or a “safer alternative”, because they do not always inhale or perceive them as addictive, the proposed cigar warning that “Cigars are not a safe alternative to cigarettes” may not be as believable as other cigar warnings [24,25,26]. Given the lack of research on cigar warnings, differences in risk perception, and a changing regulatory landscape, we conducted an experiment as part of a national phone survey of adults in the U.S. to simultaneously examine the effect of various messages and sources on the believability of cigar warnings. 

## 2. Materials and Methods

### 2.1. Sample and Measures

Data were collected as part of a nationally representative telephone survey that was conducted by the Center for Regulatory Research on Tobacco Communication [27]. Between September 2014 and May 2015, 5014 adults completed the survey (42% weighted response rate). The survey included landline and cell-phone frames and oversampled low-income respondents, individuals living in higher tobacco use regions, and young adults (age 18–25). The study was approved by University of North Carolina at Chapel Hill’s Institutional Review Board (#13-2779). For more information on survey design, survey weighting, or control variables, refer to Boynton et al. (2016) [27].

Using a three [Message] X four [Source] experimental design, each adult was randomly assigned to one of 12 cigar warning conditions. Three messages mandated by FTC at the time of the survey specifically mention cigar use; the other two cigar warning messages refer to tobacco use and do not specifically mention cigars [7]. We chose to use the cigar-specific warning messages: “Cigar smoking can cause cancers of the mouth and throat, even if you do not inhale”, “Cigar smoking can cause lung cancer and heart disease” and “Cigars are not a safe alternative to cigarettes” [7]. Three government agency sources were compared to a no source condition for the warnings: Warning (no source), Surgeon General Warning, FDA Warning, and CDC (Centers for Disease Control and Prevention) Warning. For example, for the warning including the mouth and throat message, and Surgeon General source, participants were read: “Imagine seeing this cigar warning: Surgeon General Warning. Cigar smoking can cause cancers of the mouth and throat, even if you do not inhale”.

After hearing the warning, participants were asked “How believable is this warning? Would you say not at all, somewhat, or very?”. For analysis, responses were dichotomized to “not at all” and “somewhat” versus “very” believable. Previous studies have demonstrated the importance of believability as a metric of warning label impact [21,22].

Covariates included age, sex, race/ethnicity, sexual orientation, education, poverty status (above or below poverty line), trust in federal government, any tobacco use, and awareness of CDC and FDA. Trust in federal government was classified as low if participants reported having “none at all”, “not very much”, or “no opinion” or high if participants reported “a great deal” or “a fair amount” in response to the question, “How much trust do you have in the federal government?”. Awareness of the CDC and FDA were classified as “yes” or “no” in response to the question, “Have you ever heard of the CDC or the Centers for Disease Control and Prevention?”, and “Have you ever heard of the FDA or the Food and Drug Administration?”.

Participants were classified as any current tobacco product users if they had used any of the following tobacco products in the past 30 days: e-cigarettes or other vaping devices; little cigars or cigarillos; waterpipe tobacco; cigarettes; or any other tobacco product, such as chewing tobacco, dip, snus, or premium cigars.

### 2.2. Data Analysis

The likelihood of saying that the warning was “very believable” was modeled with weighted logistic regression as a function of warning and source, controlling for covariates. In our final model, 485 observations (9.7% of the sample) were deleted because they were missing data on one or more of the explanatory variables. Results from frequency analysis and the logistic regression models include weighted percentages, adjusted odds ratios (AOR), and confidence intervals (CI). For all analyses, statistical significance was set at *p* < 0.05.

## 3. Results

Table 2 provides weighted demographic characteristics for our sample (*n* = 5014). Most participants were female (51.5%), over the age of 25 (85.1%), and non-Hispanic White (62.1%). Participants tended to have greater than a high school degree (57.4%), but our sample also included 868 (17.5%) people that reported incomes that were below the poverty line, as determined by their household size and income [27]. Slightly more than a quarter reported using any tobacco product in the past 30 days (28.4%), and 3.3% of participants identified as gay, lesbian, or bisexual. Most participants were aware of the CDC (83.6%) and FDA (94.3%), and slightly more than half reported having high trust in the federal government (57.6%).

Overall, 66.9% said the cigar warning messages were very believable. Table 3 shows the weighted logistic regression results. The warning “Cigar smoking can cause lung cancer and heart disease” was associated with higher odds of being very believable (AOR: 2.05, 95% CI: 1.55, 2.70) as compared to the message “Cigar smoking can cause cancers of the mouth and throat, even if you do not inhale”. The warning “Cigars are not a safe alternative to cigarettes” was associated with lower odds of being very believable (AOR: 0.71, 95% CI: 0.55, 0.92). Warning source (Surgeon General, FDA, CDC, no source) was not significantly associated with warning believability. There were no significant interactions between warning message and source, and interactions were removed from the final model (data not shown).

Overall, the warnings were widely believable among these sub-populations, with no differences by age, sex, sexual orientation, education, poverty status, any tobacco use, or awareness of the CDC or FDA. Being non-Hispanic “other” race when compared to being non-Hispanic white was associated with lower odds of reporting the cigar warning as very believable (AOR: 0.56, 95% CI: 0.37, 0.85). Additionally, individuals with low trust in the federal government (AOR: 0.73, 95% CI: 0.58, 0.91) had lower odds of reporting the cigar warnings as very believable, as compared to individuals with high trust.

## 4. Discussion

This study is the first experimental study among adults about currently mandated cigar warnings and the believability of the three cigar-specific warning messages mandated for cigars sold in the U.S. Our findings indicate that of the three mandated cigar warnings tested in this study, the warning that “Cigar smoking can cause lung cancer and heart disease” was significantly more believable than the warning “Cigar smoking can cause cancers of the mouth and throat, even if you do not inhale”. Both of these cigar warnings were significantly more believable than the warning “Cigars are not a safe alternative to cigarettes”. We also assessed the believability of the cigar warnings among at-risk populations, including people who are more likely to use cigars or tobacco products (men, young adults (18–25), people that identify as Black non-Hispanic or other non-Hispanic, people who identify as lesbian, gay, and bisexual, people with lower educational attainment, and people with lower income) [4]. These three cigar warnings were widely believable across each of these at-risk subgroups, and we did not observe differences in the believability by demographic characteristics. These findings highlight the importance of testing specific cigar warnings to see which might be most effective, the reasons why, and if they resonate across multiple at-risk groups. 

Differences in the believability of cigar warnings by message may be particularly important for regulators to keep in mind as they develop and evaluate current tobacco warnings for cigars. Believability is a critical indicator of warning label comprehension, affective and cognitive reactions, and impact [21,22,23]. As mandated by the FDA, cigars carry six rotating messages [10,11]. In this study, we tested three of these messages. Future research should test the believability of the remaining cigar warnings, as well as the additional impact of graphical depictions of these cigar warnings. 

The differences that were observed by cigar warning messages among adults in this experiment are similar to two other experiments about cigarette warnings on addiction [28], as well as messages about diseases recently linked to cigarettes [29]. Believability in each of these experiments seemed to be lower for messages about new information, or information that the public is misinformed about, whereas the messages that were more believable tended to be regarding information that is already public knowledge. None of these studies among adults found differences in believability by warning source. Findings are also similar to a recent publication which implemented the same experiment in a concurrent phone survey of adolescents [16]. Adolescents also found “Cigar smoking can cause lung cancer and heart disease” to be the most believable message, and there were no differences by source in the adolescent sample either. This experiment expands on the evidence offered by the previously published works by addressing cigar warnings among adults. Taken together, these studies indicate that there are important differences in the believability of warnings depending on the message across both youth and adults. 

The lack of difference in believability by source in our experiment may be due to the comparison of three different government health agencies and a no source condition. However, trust in federal government did influence the believability of warnings, such that participants with higher trust in federal government were more likely to rate warnings as highly believable. It is possible that adults perceive the source agencies in similar ways or that adults in the U.S. may assume that a tobacco warning is coming from a government health agency, even if this is not explicitly stated. Recently published data from the same dataset found that trust in CDC and FDA is 64.6% and 62.5%, respectively [30]. Previous evidence indicates for-profit sources, like the tobacco industry, can result in significantly lower believability by source [18,19,31]. 

There are a few limitations of this study. Due to the mode of our survey, participants heard the warnings over the phone, although outside of the research setting these would been seen on cigar packaging, advertisements, or at the point of sale. We did not assess use of cigars overall in our survey, however we did assess use of little cigars and cigarillos, which was not a significant predictor, and was removed from the final model. Further, the believability of the warnings was not related to the measure of any tobacco use, so believability may not differ by specific tobacco use pattern. 

## 5. Conclusions

This is the first study experimentally examining effectiveness of cigar warning messages among adults. Using a three [Message] X four [Source] experimental design, we tested cigar warnings that are currently mandated in the U.S. and varied the source associated with the warning. We found that some of the currently mandated cigar warnings in the U.S. are more believable than others, but in our experiment, warning source was not associated with believability. Further research on cigar warnings is necessary to better understand how to optimize tobacco health warnings on cigars to educate the public on health risks and prevent uptake of these products.

## Figures and Tables

**Table 1:** Cigar warnings mandated by Food and Drug Administration (FDA) deeming rule.

1	WARNING: Cigar smoking can cause cancers of the mouth and throat, even if you do not inhale.
2	WARNING: Cigar smoking can cause lung cancer and heart disease.
3	WARNING: Cigars are not a safe alternative to cigarettes.
4	WARNING: Tobacco smoke increases the risk of lung cancer and heart disease, even in nonsmokers.
5a *	WARNING: Cigar use while pregnant can harm you and your baby.
5b *	SURGEON GENERAL WARNING: Tobacco Use Increases the Risk of Infertility, Stillbirth and Low Birth Weight.
6	WARNING: This product contains nicotine. Nicotine is an addictive chemical.

* Indicates that manufacturers can choose to display either of these two warning statements.

**Table 2:** Weighted participant characteristics, *n* = 5014.

Variable	N (Weighted %)
Age	
Adult, greater than 25 years old	4205 (85.14)
Young adult, 18–25 years old	809 (14.86)
Sex	
Female	2640 (51.48)
Male	2372 (48.52)
Race/Ethnicity	
Non-Hispanic White	3280 (62.06)
Non-Hispanic Black	948 (17.74)
Non-Hispanic Other race	328 (5.90)
Hispanic	432 (14.30)
Sexual orientation	
Straight or heterosexual	4730 (96.75)
Gay, lesbian or bisexual	192 (3.25)
Education	
Greater than high school	3241 (57.42)
High school or less	1756 (42.58)
Poverty status	
Above the poverty line	3772 (82.53)
Below the poverty line	868 (17.47)
Trust in federal government	
High (a great deal, a fair amount)	2703 (57.56)
Low (none at all, not very much, no opinion)	2289 (42.44)
Any tobacco use	
No tobacco product use, past 30 days	3381 (71.57)
Any tobacco product use, past 30 days	1633 (28.43)
Awareness of the CDC	
Yes	4226 (83.64)
No	788 (16.36)
Awareness of the FDA	
Yes	4758 (94.27)
No	256 (5.73)

**Table 3:** Weighted logistic regression results.

Variable	Reported Very Believable N (Weighted %) ^a^	Very Believable vs. Not at All or Somewhat Believable AOR (95% CI)
Message		
Cigar smoking can cause cancers of the mouth and throat even if you do not inhale	1087 (66.09)	REF
Cigar smoking can cause lung cancer and heart disease	1260 (78.62)	2.05 (1.55, 2.70) *
Cigars are not a safe alternative to cigarettes	958 (56.09)	0.71 (0.55, 0.92) *
Source		
No source	779 (67.45)	REF
Surgeon General	838 (66.96)	0.98 (0.72, 1.35)
FDA	851 (68.20)	1.10 (0.80, 1.52)
CDC	837 (64.94)	0.87 (0.63, 1.21)
Age		
Adult, greater than 25 years old	2795 (67.83)	REF
Young adult, 18–25 years old	510 (61.39)	0.94 (0.70, 1.27)
Sex		
Female	1742 (68.21)	REF
Male	1562 (65.43)	0.98 (0.77, 1.23)
Race/Ethnicity		
Non-Hispanic White	2171 (69.54)	REF
Non-Hispanic Black	641 (62.33)	0.75 (0.55, 1.03)
Non-Hispanic Other race	195 (61.20)	0.56 (0.37, 0.85) *
Hispanic	283 (62.16)	0.77 (0.54, 1.08)
Sexual orientation		
Straight or heterosexual	3140 (67.27)	REF
Gay, lesbian or bisexual	117 (64.47)	0.93 (0.58, 1.49)
Education		
Greater than high school	2175 (66.55)	REF
High school or less	1116 (67.12)	1.11 (0.86, 1.44)
Poverty status		
Above the poverty line	2533 (68.50)	REF
Below the poverty line	541 (63.56)	0.90 (0.65, 1.26)
Trust in federal government		
High	1859 (69.10)	REF
Low	1432 (63.76)	0.73 (0.58, 0.91) *
Any tobacco use		
No tobacco product use, past 30 days	2322 (68.32)	REF
Any tobacco product use, past 30 days	983 (63.21)	0.88 (0.68, 1.14)
Awareness of the CDC		
Yes	2808 (67.75)	REF
No	497 (62.31)	0.96 (0.68, 1.35)
Awareness of the FDA		
Yes	3152 (67.73)	REF
No	153 (52.60)	0.58 (0.34, 1.00)

Notes: **^a^**. The percent who reported “very believable” refers to the pooled messages, rather than a specific message; * indicates significance at *p* < 0.05.
